# Epithelial-mesenchymal transition induced by GRO-α-CXCR2 promotes bladder cancer recurrence after intravesical chemotherapy

**DOI:** 10.18632/oncotarget.16786

**Published:** 2017-04-03

**Authors:** Lu Chen, Xiu-Wu Pan, Hai Huang, Yi Gao, Qi-Wei Yang, Lin-Hui Wang, Xin-Gang Cui, Dan-Feng Xu

**Affiliations:** ^1^ Department of Urinary Surgery of Ruijin Hospital, Shanghai Jiaotong University, Shanghai 200025, China; ^2^ Department of Urinary Surgery of Third Affiliated Hospital, Second Military Medical University, Shanghai 201805, China; ^3^ Department of Urinary Surgery of Changzheng Hospital, Second Military Medical University, Shanghai 200003, China

**Keywords:** GRO-α, CXCR2, bladder cancer, epithelial-mesenchymal transition, recurrence

## Abstract

Non-muscle invasive bladder cancers (NMIBC) are typically treated by transurethral resection with intravesical chemotherapy. However, the post-therapeutic incidence of tumor recurrence and progression to muscle invasive disease is high, and the underlying mechanism(s) remains unknown. In this study, we observed that recurrent bladder cancer cells exhibit a mesenchymal phenotype, which is initiated by the autocrine GRO-α signaling. Mechanically, the chemotherapeutic drug epidoxorubicin induces GRO-α expression in primary bladder cancer cells at G1/S phase via p38-dependent activation of NF-κB. GRO-α phosphorylation of Snail on Ser246 supports Snail's accumulation in the nucleus, and thereby promotes transcription repression activity of Snail from E-cadherin promoters. In accordance, disrupting the GRO-α-Snail axis in NMIBC represents a promising alternative to prevent post-therapeutic tumor progression and recurrence.

## INTRODUCTION

Approximately 75–85% cases of newly diagnosed bladder cancer are non-muscle invasive bladder cancer (NMIBC), including non-muscle invasive papillary or flat lesions (pTa and pTis, respectively) and invasive urothelial carcinomas involving the lamina propria (pT1) [1]. NMIBC is typically treated by transurethral resection and intravesical chemotherapy. The American Urological Association (AUA) considers all high grade pTa, pTis and pT1 to be at high risk for recurrence and progression; pT1 tumors in particular show a highly divergent behavior, with progression rates of 17% to 50%, despite therapy. Muscle-invasive bladder carcinoma is clinically unfavorable, with a 5-year overall survival rate of 48% to 67% [2, 3]. Therefore, development of novel therapies is needed to maximize the benefits of treatment, notably reducing disease recurrence and preventing progression.

Tumor cells progress from non-invasive to malignant phenotypes via a series of critical steps that involve morphological changes referred to as the epithelial–mesenchymal transition (EMT). EMT is a process originally observed during the embryonic development, in which cells lose epithelial characteristics [4, 5]. Activation of the latent EMT program confers upon cancer cells a distinct advantage for local invasion [6, 7]. In deed, Baumgart et al. characterized the expression of E-cadherin, β-catenin, plakoglobin, and vimentin in a series of primary bladder tumors. Downregulation of epithelial markers was associated with disease progression in terms of both grade and stage (i.e., superficial vs. muscle-invasive cancer), and in univariate analyses, downregulation of either β-catenin or plakoglobin was associated with shorter disease-specific survival [8]. With respect to the chemotherapy, previous studies have demonstrated a close link between EMT and treatment of chemotherapeutic agents. For example, Li et al. previously showed that transient adriamycin treatment induced EMT and apoptosis simultaneously in breast cancer cells. Only the cells undergoing EMT showed enhanced invasion/metastasis and multidrug resistance [9]. In addition, Kajiyama et al. identified an association between chronic paclitaxel resistance and induction of EMT in epithelial ovarian carcinomas [10]. Based on these observations, we hypothesize that EMT may contribute to the malignant phenotypes of bladder cancers after receiving chemotherapy.

In the current study, we demonstrated that recurrent bladder cancer cells undergoing chemotherapy preferentially exhibited a mesenchymal phenotype. In particular, recurrent bladder cancer cells undergoing chemotherapy secret growth-regulated protein alpha (GRO-α), which triggers the process of EMT through regulating phosphorylation and functions of Snail. In clinical settings, treatment of relapsed tumor using the regimen that resulted in remission of the primary tumor is suboptimal. The current study raises the possibility of preventing/delaying tumor relapse via manipulating specific signaling pathway(s).

## RESULTS

### Recurrent bladder cancer cells exhibit a mesenchymal phenotype

**Figure 1:**
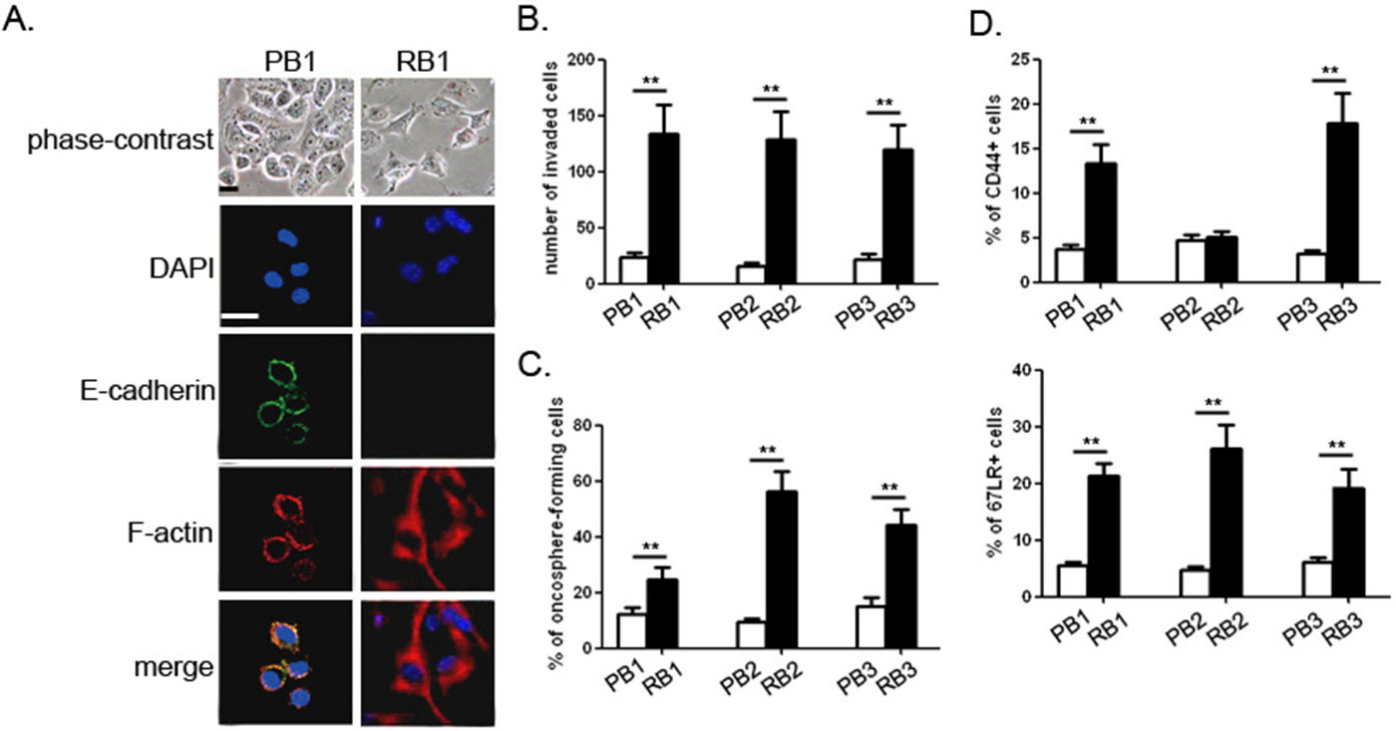
Recurrent bladder cancer cells exhibit a mesenchymal phenotype **(A)** Morphology examination and immunofluorescence of E-cadherin, F-actin in paired primary and relapsed bladder cancer cells. **(B)** Invasive rate of the paired primary and relapsed bladder cancer cells. **(C)** Dissociated cells from the indicated tumor samples were incubated in the presence of growth factors for 7 days. The percentage of oncosphere-forming cells was determined. **(D)** The percentage of CD44- and 67LRe-positive cells were examined in the paired primary and recurrent bladder carcinomas by flow cytometric analysis. Experiments were performed three times independently, and results are shown as mean±SD. **, p<0.05 versus the corresponding controls. Bars: 10 μm.

We obtained bladder cancer cells from three paired primary and relapsed tumor samples (PB1-3 *vs*. RB1-3). Routine morphologic observation showed that primary tumor cells grew in the form of tightly-packed colonies characteristic of epithelial cells, while most recurrent tumor cells looked flattened, spread actively and underwent cytoskeletal rearrangement (Figure 1A). These phenomena were associated with decreased expression of E-cadherin, formation of actin stress fibers (F-actin), and up-regulation of N-cadherin (Figure 1A and Supplementary Figure 1A). Compared with their corresponding primary counterparts, the invasion rate of the recurrent tumor cells increased by 5.7-8.2 fold (Figure 1B), without showing any change in cell proliferation(Supplementary Figure 1B).

EMT is a process reminiscent of CSCs. Within 7 days, all individual cells produced non-adherent multi-cellular oncospheres highly expressing bladder CSC markers, such as CD44 and 67LR (Supplementary Figure 1C). Noticeably, the recurrent bladder cancer cells showed a 2.1-5.8-fold increase in oncosphere-forming capacity over the matched primary tumor cells (Figure 1C). Moreover, we performed limiting dilution assays to determine self-renewal capacity. Oncospheres from primary tumors showed limited expansion and could not be maintained beyond passage 2 (<2 passages), while all recurrent tumors yielded sphere cells with extended passages (>4 passages). To extend these observations, we examined the expression of putative CSC markers in the paired primary and recurrent bladder carcinomas by flow cytometric analysis. As compared with the matched primary tumors, most recurrent bladder cancer cells (RB1 and RB3) possessed a higher percentage of CD44- and 67LR-positive populations. Although there was only a negligible difference in the amount of CD44-positive cells between PB2 and RB2, RB3 possessed a 2.3-fold increase in 67LR-positive population compared with PB3 (Figure 1D). These data suggest that recurrent bladder cancer cells underwent epithelial-mesenchymal transition accompanied with malignant progression.

### GRO-α derived from recurrent bladder cancer cells elicits epithelial-mesenchymal transition

**Figure 2:**
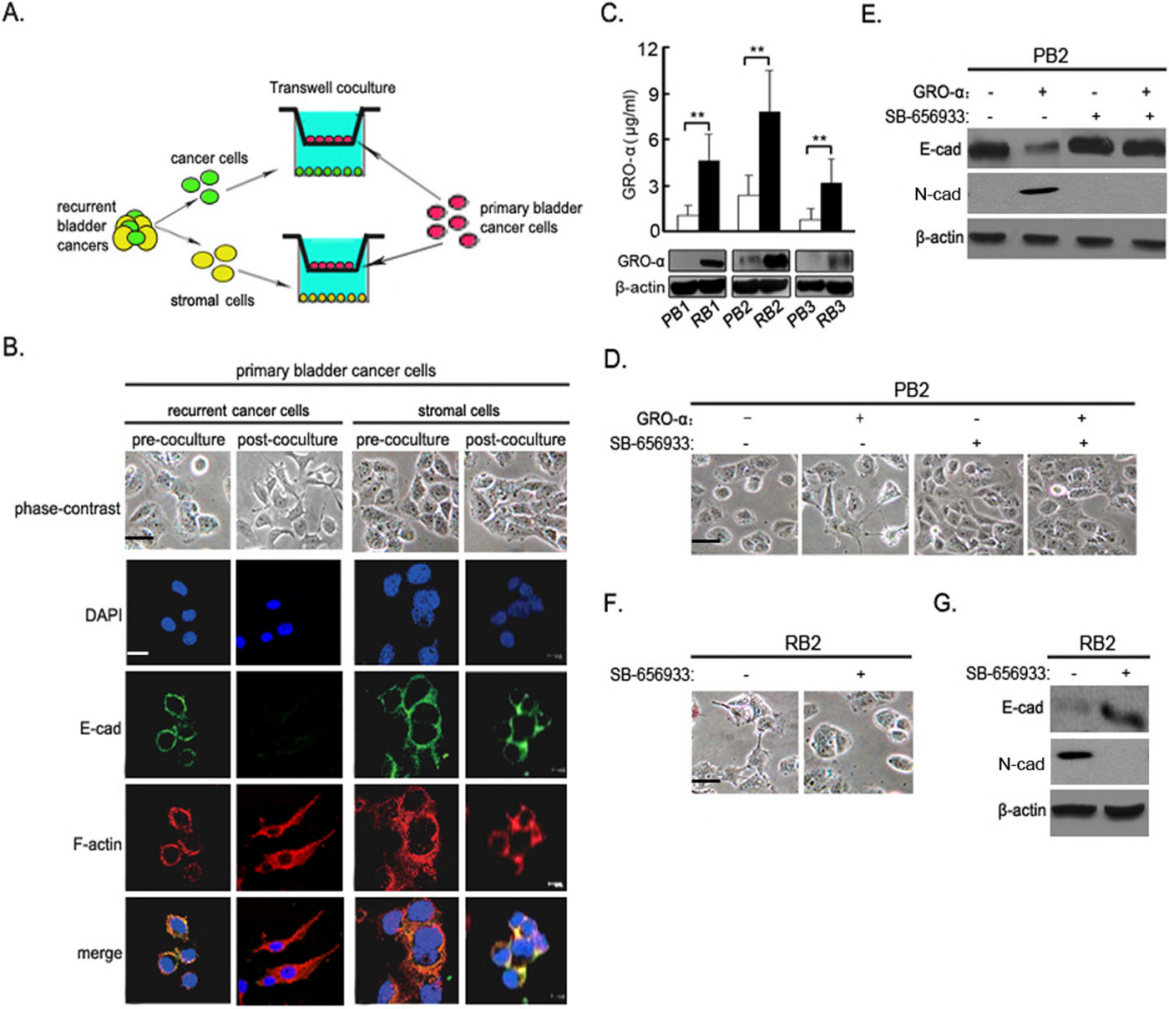
GRO-α derived from recurrent bladder cancer cells elicits epithelial-mesenchymal transition **(A)** Cartoon depicting the experimental approach adopted to for the coculture Transwell system. **(B)** After PB1 cells were transwell-cultured with RB1 or stromal cells as described in **(A)**, cell morphology and immunofluorescence of E -cadherin, F-actin were examined. **(C)** GRO-α secretion in supernatant, as well as its expression in cell lysates, in the recurrent cancer cells was evaluated by ELISA and western blot analysis, respectively. **(D, E)** PB2 cells were incubated with 5μg/ml GRO-α and/or 1μM SB656933 for 72h, after which cell morphology **(D)** and the expression level of E-cadherin, N-cadherin **(E)** were examined. **(F, G)** RB2 cells were incubated with 1μM SB656933 for 72h, after which cell morphology **(F)** and the expression level of E-cadherin, N-cadherin **(G)** were examined. Experiments were performed three times independently, and results are shown as mean±SD. **, p<0.05 versus the corresponding controls. Bars: 10 μm.

To figure out whether the differential phenotypes were attributed to cancer cells themselves or the unique microenvironment, we isolated epithelial and stromal cells from recurrent tumor tissues and transferred them to the upper chamber of the Transwell, while the bottom compartment was seeded with the matched primary tumor cells (Figure 2A). This transwell system allows for exchange of diffusible factors but not cells between the chambers. PB1 cells cocultured with RB1 cells showed a fibroblast-like cell morphology. Furthermore, coculture with RB1 cells induced the loss of E-cadherin expression and formation of actin stress fibers in PB1 cells (Figure 2B). Correspondingly, increased expression of N-cadherin and down-regulation of E-cadherin were also observed in PB1 cells (Supplementary Figure 2A). In contrast, no similar phenomena were observed in PB1 cells cocultured with stromal cells (Figure 2B and Supplementary Figure 2A), suggesting that recurrent bladder cancer cells themselves created a distinct environment for induction of EMT.

Using SINQ and MaxQuant label-free proteomic software, we performed a comparative analysis on differentially expressed proteins in the supernatant of recurrent bladder cancer cells. As compared with PB1 cells, 23 proteins in RB1 cells underwent differential expression by at least 3 fold, among which GRO-α underwent more than five-fold change. We further validated GRO-α expression, and found that GRO-α secretion, as well as its expression, in the recurrent cancer cells was significantly elevated as compared with that in their primary counterparts (Figure 2C). CXCR2 is the specific receptor for GRO-α. Recurrent bladder cancer cells exhibited similar levels of CXCR2 in comparison with the corresponding primary cancer cells (Supplementary Figure 2B). These data suggest the existence of autocrine GRO-α-CXCR2 signaling in recurrent bladder cancer cells.

Next, we evaluated the effect of GRO-α on EMT in bladder cancer cells. Following GRO-α treatment for 24h, primary PB2 cells exhibited a marked alteration in cell morphology, changing from the characteristic organized ‘cobblestone’ appearance of epithelial cell monolayers to a disorganized elongated fibroblast-like phenotype. In contrast, these cells treated with the specific CXCR2 inhibitor, SB-656933, in the presence of GRO-α maintained their epithelial phenotype (Figure 2D). Moreover, treatment of RB2 cells with SB-656933 led to morphological changes consistent with transition from a mesenchymal to an epithelial phenotype (Figure 2F). Consistent with the morphological findings, immunoblotting analysis of primary PB2 revealed diminished levels of E-cadherin and increased N-cadherin expression following GRO-α treatment. In PB2 cells treated with SB-656933 and GRO-α, the expression of both E-cadherin and vimentin remained unchanged, as compared with the control cells treated with the vehicle for both conditions (Figure 2E). As anticipated, in RB2 cells, inhibition of GRO-α-CXCR2 signaling by SB-656933 potently upregulated the expression level of E-cadherin, while downregulated the mesenchymal marker N-cadherin (Figure 2G). These data suggest that mesenchymal transformation of recurrent bladder cancer cells was dependent on the autocrine GRO-α-CXCR2 signaling.

### Epidoxorubicin induces GRO-α expression in bladder cancer cells in a cell cycle-dependent manner via the p38/MAPK-NF-κB pathway

**Figure 3:**
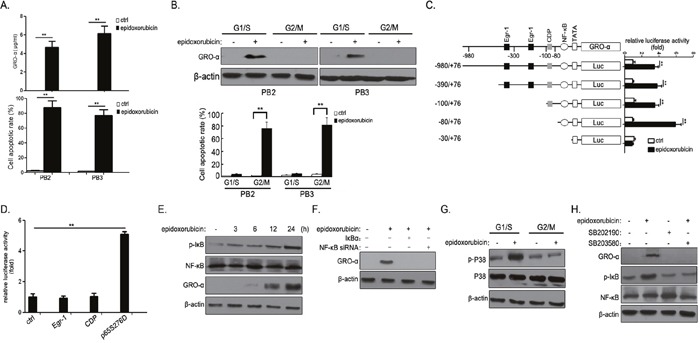
Epidoxorubicin induce GRO-α expression in bladder cancer cells in a cell cycle-dependent manner via the p38/MAPK-NF-κB pathway **(A)** PB2 and PB3 cells were incubated with 1μg/ml epidoxorubicin for 48h, after which GRO-α secretion and cell apoptotic rate were assessed by ELISA and flow cytometry, respectively. **(B)** PB2 and PB3 cells were synchronized in G1/S boundary by the addition of 2mmol/l hydroxyurea to the medium for 16h. G2/M synchronization was achieved by maintaining the cells inculture with 0.1ng/ml nocodazole for 20h. After being synchronized to G1/S or G2/M phase, cells were treated with 1μg/ml epidoxorubicin for another 48h to assess GRO-α expression or apoptotic rate. **(C)** PB2 cells were transiently transfected with 0.2μg of a series of 5′-deletion constructs of human GRO-α promoter reporter plasmids. Forty-eight hours later, the cells were either untreated or treated with 1μg/ml epidoxorubicin for 8h, and luciferase activities were measured. **(D)** PB2 cells were transiently co-transfected with 0.2μg of pGRO-α-Luc(−980/+76) and expression plasmids for Egr-1 (pCDNA3.1/Egr-1), CDP (pCDNA3.1/CDP) or p65S276D (pCDNA3.1/p65S276D). After 48h, the cells were collected and assayed for luciferase activity. **(E)** PB2 cells were treated with 1μg/ml epidoxorubicin for the indicated times, after which the expression level of phospho-IκB, NF-κB and GRO-α was determined. **(F)** PB2 cells were transiently transfected with expression plasmids for IκBα or siRNA against NF-κB. After 48h, the cells were treated with 1μg/ml epidoxorubicin for another 48h and the expression level of GRO-α was examined. **(G)** After being synchronized to G1/S or G2/M phase as described in **(B)**, PB1 cells were treated with 50μg/ml epidoxorubicin for another 48h to assess the expression level of phosphor-P38 and P38. **(H)** PB1 cells were treated with 1μg/ml epidoxorubicin or/and 5μM SB202190 or 10μg/ml SB203580 for 48h, after which the expression level of GRO-α, phospho-IκB and NF-κB was determined. Experiments were performed three times independently, and results are shown as mean±SD. **, p<0.05 versus the corresponding controls.

The bladder cancer patients in our series experienced recurrence after intravesical chemotherapy. Epidoxorubicin, the drug used most often for intravesical chemotherapy, induced concurrence of different events (apoptosis *vs*. GRO-α secretion) in PB2 and PB3 cells (Figure 3A). To investigate the underlying mechanism, we examined the contribution of cell cycle state. PB2 and PB3 cells were synchronized at either G1/S or G2/M phase, after which epidoxorubicin-induced effects were examined. As shown in Figure 3B, epidoxorubicin induced GRO-α expression only in cells synchronized at G1/S phase, whereas it induce apoptosis in cells synchronized at G2/M phase.

We then investigated potential transcription factors involved in regulation of GRO-α expression. The GRO-α promoter 5′-flanking region (spanning from bp −980 to +76) and its sequentially deleted fragments (starting from −980, −390, −100 and −80 to +76bp) were prepared by PCR and cloned into luciferase expression vectors (Figure 3C). Stimulation with epidoxorubicin markedly increased the luciferase activity in constructs containing sequences from −980 to −80bp, which included NF-κB binding sites. However, the construct from which the sequence from −980 to −80bp was deleted produced no response to drug treatment. The construct lacking the sequence from −980 to −100bp produced the strongest luciferase activity in drugs-stimulated cells (Figure 3C), suggesting the involvement of NF-κB in regulating GRO-α expression. To test this possibility, we evaluated the GRO-α promoter activity after cotransfection of the candidate transfactor in primary bladder cancer cells. It was found that the expression of p65 S276D (a constitutively active form of NF-κB) was associated with a significant increase in the GRO-α promoter activity, while Egr1, CDP had no effect on promoter activity in the context of primary bladder cancer cells (Figure 3D). The crucial event in the activation of NF-κB is the phosphorylation of IκB. In agreement with this, IκB phosphorylation was induced 3h after epidoxorubicin treatment, earlier than GRO-α expression (Figure 3E). The drug-induced GRO-α expression was significantly suppressed by either IκBα super-repressor (a protein with mutations that render it resistant to stimulus-induced degradation) or NF-κB siRNA in PB2 cells (Figure 3F).

Previous studies indicated that MAPK/p38 activation promotes cancer cell survival after chemotherapy [11, 12]. Epidoxorubicin induced p38 activation in PB1 cells at G1/S phase but not at G2/M phase (Figure 3G). P38 signaling inhibition with SB202190 or SB203580 showed that IκB phosphorylation was p38 dependent, as p38 inhibition led to attenuation in drug-induced phospho-IκB abundance and subsequent GRO-α expression (Figure 3H). These data suggest that epidoxorubicin induced GRO-α expression in primary bladder cancer cells at G1/S phase via p38-dependent activation of NF-κB.

### GRO-α-induced PI3K-mediated phosphorylation of Snail modulates Snail's subcellular localization and functions

**Figure 4:**
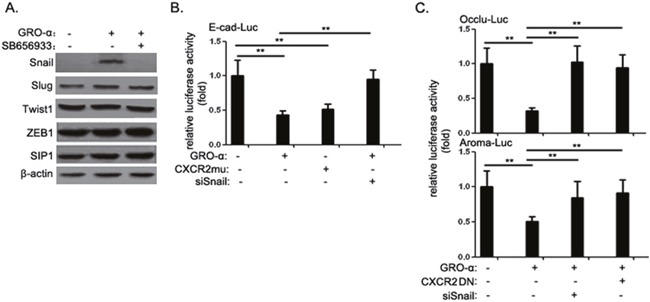
The GRO-α-CXCR2 axis promotes Snail expression and its repressor activity **(A)** PB2 cells were treated with 1μg/ml epidoxorubicin or/and 1μM SB656933 for 24h, after which the expression level of Snail, Slug, Twist1, ZEB1 and SIP1 was assessed by western blot analysis. **(B)** E-cadherin luciferase reporter assay: 100ng of luciferase reporter constructs carrying human E-cadherin promoter were transfected into PB2 cells singly or together with CXCR2mu expression vector or cotransfection of Snail siRNA, respectively; After treatment with or without 5μg/ml GRO-α for 24h, the cells were collected and assayed for luciferase activity. **(C)** Occludin and aromatase luciferase reporter assay: 100ng of luciferase reporter constructs carrying human occludin and aromatase promoter were transfected into PB2 cells singly or together with CXCR2DN expression vector or cotransfection of Snail siRNA, respectively; After treatment with or without 5μg/ml GRO-α for 24h, the cells were collected and assayed for luciferase activity. Experiments were performed three times independently, and results are shown as mean±SD. **, p<0.05 versus the corresponding controls.

In order to identify which GRO-α response mediated EMT, we examined the effect of GRO-α or/and SB656933 on expression of various transcription factors for EMT. In PB3 cells, snail expression was induced by GRO-α after 24h treatment and significantly suppressed by pre-treatment of SB656933. However, the levels of other EMT-related molecules, such as slug, twist1, ZEB1 and SIP1, were not affected (Figure 4A). To explore the potential effect of GRO-α signaling upon the functions of Snail, we examined the impact of GRO-α on the ability of Snail to repress E-cadherin promoter activity. Our results showed that either GRO-α or constitutively active CXCR2 mutant had an inhibitory effect on E-cadherin promoter activity. GRO-α-induced effects were largely abolished by knockdown of Snail (Figure 4B). We also examined the effect of GRO-α on two additional Snail-regulated genes, tight junction membrane protein occludin and estrogen synthetase enzyme aromatase. GRO-α treatment promoted the ability of Snail to repress transcription from both occludin- and aromatase-promoter reporters. Consistent with the role of GRO-α in modulating Snail activity, inhibition of GRO-α-CXCR2 signaling with a dominant-negative CXCR2 relieved the repression effect of Snail on occludin and aromatase promoter activity (Figure 4C).

**Figure 5:**
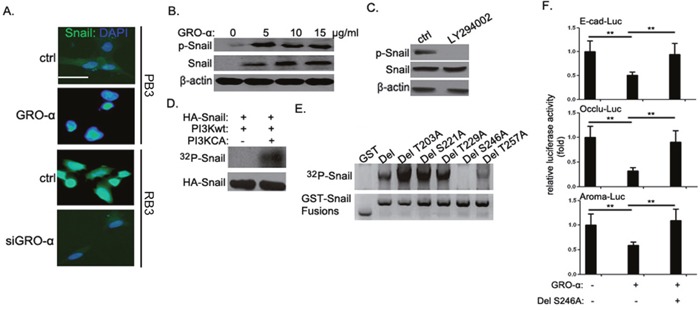
GRO-α regulation of Snail phosphorylation and subcellular localization **(A)** Confocal microscopic analysis of Snail localization in RB3 cells transfected with GRO-α siRNA or inPB3 cells treated with 5μg/ml GRO-α for 36h. **(B)** PB3 cells were treated with the indicated concentrations of GRO-α for 36h, after which the expression level of phospho-Snail and Snail were assessed. **(C)** RB3 cells were treated with 50μg/ml LY294002 for 48h, after which the expression level of phospho-Snail and Snail were assessed. **(D)** 293 T cells were serum-starved for 1 day and then transfected with Snail expression vector together with either wild-type PI3K or constitutively active form of PI3K (PI3KCA). Cells were labeled with [32P]orthophosphoric acid and cell lysates were immunoprecipitated with an anti-HA mAb. Phosphorylation was visualized by autoradiography with a PhosphoImager. **(E)** *In vitro* phosphorylation of Snail deletion constructs by GRO-α. All five different point mutation constructs were made from a Snail deletion construct (amino acids 181-264). **(F)** E-cadherin, occludin and aromatase luciferase reporter assay: 100ng of luciferase reporter constructs carrying human E-cadherin, occludin and aromatase promoter were transfected into PB3 cells singly or together with Snail DelS246A expression vector; After treatment with or without 5μg/ml GRO-α for 24h, the cells were collected and assayed for luciferase activity. Experiments were performed three times independently, and results are shown as mean±SD. **, p<0.05 versus the corresponding controls. Bar: 5μm.

To gain deeper insights into the regulatory effect of GRO-α-CXCR2 signaling on Snail functions, we examined the effect of GRO-α upon the subcellular localization of Snail in bladder cancer cells by scanning confocal microscopy. We found that knockdown of GRO-α expression by siRNA led to almost complete disappearance of nuclear Snail in RB3 cells, while GRO-α promoted nuclear translocation of cytoplasmic Snail in PB3 cells (Figure 5A). Interestingly, we noticed dose-dependent phosphorylation of Snail by GRO-α (Figure 5B). PI3K and p38 MAPK are two important downstream signaling pathways of CXCR2 [13–15]. Inhibition of PI3K pathway, but not p38 signaling, largely abolished GRO-α-induced Snail phosphorylation in the RB3 cells (Figure 5C and Supplementary Figure 3A). To determine whether Snail was a novel substrate of PI3K, we then did the *in vivo* Snail phosphorylation assay. We cotransfected serum-starved 293 T cells with HA-tagged Snail, with or without catalytically active PI3K (PI3KCA), after which the cells were labeled with [32P]orthophosphoric acid. It was found that HA-Snail could be phosphorylated by PI3KCA but not by wild type PI3K in serum-starved conditions (Figure 5D). To map the PI3K phosphorylation site in Snail, we identified five potential phosphorylation sites in Snail and substituted all potential serine or threonine residues with alanine in the potential phosphorylation sites by site-directed mutagenesis (Thr203Ala, Ser221Ala, Thr229Ala, Thr257Ala, and Ser246Ala) (Supplementary Figure 3B). The site of GRO-α-induced PI3K-mediated phosphorylation on Snail was identified as Ser246 (Figure 5E). Next, we compared the repression activity of Snail Ser246Ala mutant with that of wild-type Snail against a Snail-target gene reporter assay. GRO-α-mediated repression of transcription from the E-cadherin, occludin and aromatase promoters were significantly relieved by the mutant Snail Ser246Ala (Figure 5F). These results suggest that GRO-α-induced phosphorylation of Snail modulated its transcriptional activity by increasing accumulation of Snail in the nucleus.

### Overcoming post-therapeutic recurrence of bladder cancers by disrupting GRO-α-mediated EMT

**Figure 6:**
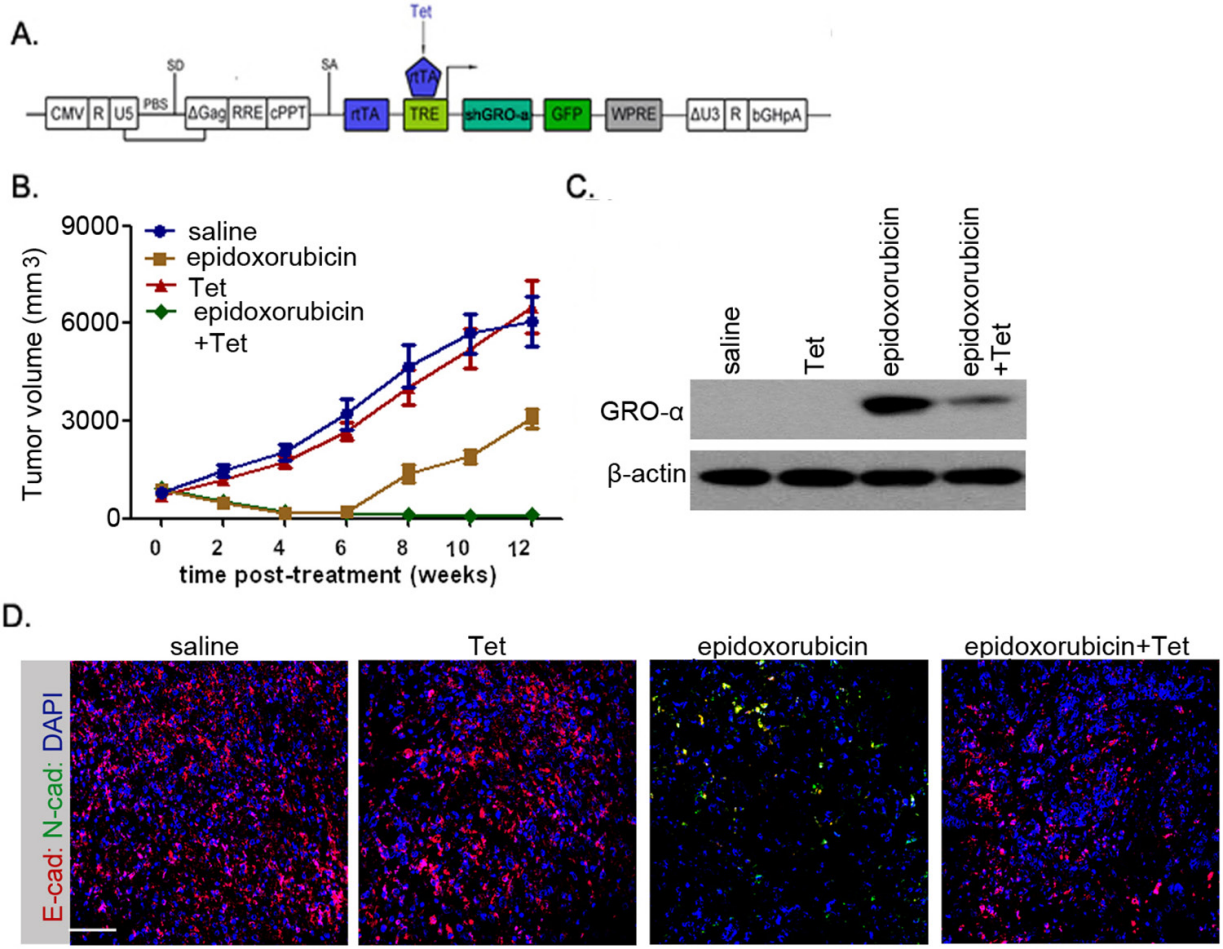
Interrupting the GRO-α-CXCR2 axis prevents EMT and abolishes post-therapeutic recurrence in bladder cancers **(A)** Schematic representation of the Tet-on lentiviral vector used in this study. PB2 cells transfected with the lentiviral vector in **(A)** were subcutaneously implanted into NOG mice and allowed to form obvious tumors in 4 weeks. Mice were randomized into groups (n=15 each) that received saline, epidoxorubicin (0.04mg/kg), Tet (4mg/kg) or epidoxorubicin+Tet. All treatments were repeated twice weekly for four weeks. **(B)** Mice were monitored weekly for tumor volume at the indicated times. **(C)** After cessation of the treatments, the expression level of GRO-α in residual tumors of each group was assessed. **(D)** Immunofluorescence of E-cadherin and N-cadherin in residual tumors of each group after cessation of the treatments. Bars: 50 μm.

Due to the significance of EMT in cancer progression, we tested whether abrogating the process of EMT would prevent post-therapeutic recurrence. We generated the PB2 cells expressing GRO-α shRNA under the conditional control of Tet (Figure 6A), after which these cells were subcutaneously transplanted into NOG mice. All 8-week-old mice harboring non-invasive bladder cancer cells were subjected to epidoxorubicin alone, or Tet alone, or epidoxorubicin plus Tet treatment. Treatment with epidoxorubicin alone produced >82% tumor regression within 4 weeks (Figure 6B), but significantly increased the percentage of N-cad+ cells, accompanied by reduced quantity of E-cad+ cells, in residual lesions (Figure 6D). Recurrence was observed in 9 out of 10 such mice within 2 weeks of treatment cessation (Figure 6B). In stark contrast, blocking GRO-α production by administration of Tet significantly abolished the process of EMT (Figure 6C and 6D). Treatment with epidoxorubicin plus Tet effectively decreased tumor size, and no recurrence was noted in any subject (Figure 6B).

## DISCUSSION

Bladder cancer carries a high risk of recurrence and poor prognosis due to muscle invasion and metastasis. In this study, we observed that bladder cancer recurrence was associated with the aberrant mesenchymal phenotype. Mesenchymal recurrent bladder cancer cells secreted multiple cytokines and growth factors, including transforming growth factor beta, interleukin-8, EGF, Wnt, and granulocyte-macrophage colony-stimulating factor, many of which are involved in malignancy progression in various types of carcinomas [16–18]. In particular, mesenchymal recurrent BCa cells undergoing chemotherapy are the major source of GRO-α. For cells at G1/S phase, chemotherapy drugs induced the expression of GRO-α. This was not observed in cells at G2/M phase. Our data well explain how bladder cancer cells respond to chemotherapy heterogeneously in terms of cell cycle phases. GRO-α is a member of the CXC chemokine family. The biological function of GRO-α is primarily mediated via CXCR2, a seven-transmembrane G protein-coupled receptor. GRO-α has been reported to play a critical role in tumorigenesis, angiogenesis, and metastasis [13–15, 19, 20]. Although a higher expression of GRO-α was associated with the invasive phenotype of bladder cancer both *in vitro* and *in vivo* [21, 22], the detailed mechanism of this chemokine in invasive potential of bladder cancer is poorly understood. As the GRO-α receptor CXCR2 is expressed in all bladder cancer cells irrespective of the mesenchymal phenotype, it is probable that secretion of GRO-α is associated with bladder cancer recurrence via an autocrine loop involving its receptor. Here, we demonstrated that the interaction between GRO-α and CXCR2 induces activation of PI3K signaling. GRO-α-induced PI3K-mediated phosphorylation of Snail on Ser246 promotes Snail's nuclear accumulation and consequently its repressor activity on the promoter of E-cadherin. Ultimately, the bladder cancer cells undergo EMT and become highly invasive. In line with these, our *in vivo* studies demonstrated that interfering with the GRO-α/CXCR2 axis considerably disrupted the process of EMT, suggesting that the GRO-α/CXCR2 axis represents the driving force of mesenchymal transformation. Mounting evidence has implied the contributions of EMT in the emergence of treatment failure and tumor recurrence. Accordingly, abolishing GRO-α-induced EMT successfully prevented post-chemotherapeutic relapse. In this sense, disrupting the GRO-α-CXCR2 axis in NMIBC may represent a promising alternative to prevent post-chemotherapy cancer progression and recurrence.

## MATERIALS AND METHODS

### Ethics statement

This study was approved by the institutional review board of Changzheng Hospital of the Second Military Medical University (Shanghai, China) and written informed consent was obtained from all patients who provided bladder cancer tissues.

### Cell culture and reagents

Tissues were harvested from bladder specimens removed at the time of Transurethral resection of bladder tumor (TURBT) for primary and recurrent carcinoma of the bladder in the same patients. Cells were cultured at 37°C with 5% CO2 in RPMI 1640 supplemented with 10% v/v Fetal bovine serum (FBS) and penicillin (100 units/ml) /streptomycin (0.1 mg/ml). Recurrent bladder tumor cultures from three patients were sorted as cancer cells and stromal cells by CK7 antibody via FACS LSRII (BD Biosciences). Cancer cells were termed as PB1, PB2 and PB3 in primary cancers, and RB1, RB2 and RB3 in recurrent cancers. 293T cells were culture at 37°C with 5% CO2 in RPMI 1640 supplemented with 10% v/v FBS and penicillin (100units/ml)/streptomycin (0.1mg/ml). SB656933, SB202190, SB203580 and LY294002 were purchased from Calbiochem (San Diego, CA, USA) and Human recombinant GRO-α was from ProSpec Biology (East Brunswich, NJ, USA). The firefly and Renilla Dual-Glo™ Luciferase Assay System was purchased from Promega (Madison, WI, USA). The pRL-null plasmid, which encodes Renilla luciferase, was purchased from Promega.

### Xenograft studies

Subcutaneous xenograft tumor models were established based on 4-week-old female BALB/c-nu mice. The mice were housed in a pathogen-free facility at the Second Military Medical University. All animal protocols are in compliance with ethical regulations and have been approved by the Institutional Animal Care Use Committee of Second Military Medical University. For Sub-Q xenografts, the indicated cells suspended in 100μl Hanks buffered saline solution were injected subcutaneously in the axillary region of the right chest in BALB/c-nu/nu mice. Tumor volume was measured every 2 days starting from day 4 post-injection to monitor the extent of tumor growth, regression and recurrence. Tumor volume was calculated using the formula: 1/2(Length×Width2). When tumor reached ∼1cm in diameter, mice were randomly divided into the indicated groups and the mean tumor volumes of each group were similar before the treatment started. No mice were excluded after the treatment started. To determine the effects of epidoxorubicin treatment alone or epidoxorubicin plus tetracycline (Tet) treatment, tumor-bearing mice were treated with intraperitoneal (i.p.) epidoxorubicin (0.04mg/kg; Sigma-Aldrich, Dorset, UK) or/and Tet (4mg/kg; Sigma-Aldrich, Dorset, UK), twice weekly for 4 weeks.

### Immunofluorescence

Cells were washed with PBS twice, fixed with 4% formaldehyde for 15 min, permeabilized with 0.1% Triton X-100/PBS for 5 min, and blocked by 3% bovine serum albumin for 30 min at room temperature. The coverslips were stained with primary antibodies overnight at 4°C followed by incubation with secondary antibody for 30 min at 37°C. Cells were washed three times with PBS in a dark chamber. The coverslips were washed as described above, inverted, mounted on slides using DAPI (Life Technologies), and examined under a Leica confocal microscope.

### Western blot analysis

Protein preparations from the indicated cells were obtained by lysing samples in 50 mmol/L of TRIS (pH 7.5), 100 mmol/L of NaCl, 1% NP40, 0.1% Triton, 2 mmol/L of EDTA, 10 Ag/mL of aprotinin, and 100 Ag/mL of phenylmethylsulfonyl-fluoride. Prestained molecular weight standards were from Bio-Rad (Milan). Proteins separated on the polyacrylamide gels were blotted on a PVDF membrane. The membrane was stained with Ponceau S (Sigma) to enable us to evaluate the success of transfer, and to locate the molecular weight markers. Free protein binding sites on the PVDF membrane were blocked with nonfat dry milk and a Tween 20/TBS solution. The membranes were washed and stained with specific primary antibodies and with secondary antisera, and then conjugated with horseradish peroxidase (Sigma-Aldrich) diluted 1:2,000. The anti-E-cadherin, anti-N-cadherin, anti-CXCR2, anti-phospho-P38, anti-P38, anti-IκB, anti-NF-κB, anti-slug, anti-twist1, anti-ZEB1, anti-Snail and anti-SIP1 antibodies were purchased from Abcam, and the anti-GRO-α antibody was from Santa Cruz. The luminescent signal was visualized with the ECL Western blotting detection reagent kit (Amersham) and quantified by scanning with a Discover Pharmacia scanner equipped with a Sun Spark Classic Workstation.

### Isolation of primary epithelial and stromal cells from bladder specimens

Tumor specimens were processed within 1 to 2 h of surgery. Each sample was minced in small fragments (<1 mm3), in a sterile environment. Tissue fragment were then digested at 37°C, for 2 to 4 h in a solution of type IV collagenase, 1 mg/ml (Sigma-Aldrich), containing 40 mg/ml of bovine serum albumin, 2 mg/ml of glucose, 100 units/ml of penicillin and 100 mg/ml of streptomycin (P+S; Sigma-Aldrich), 50 mg/L of gentamicin (Sigma-Aldrich), and 1.25 mg/L of Fungizone (Life Technologies). After incubation, the samples were extensively rinsed with PBS and suspended in culture media. Each sample was divided into two aliquots, one to be frozen and one to be cultured. Cells underwent three cycles of centrifugation and were differentially separated by centrifugation. Briefly, increasing speeds of centrifugation (40-100-200×g) generated three cell populations: epithelial-enriched (EE), stromal-enriched (SE), and organoid substance (OS). The cells were seeded separately in 24-wells at a density of 105 cells/well and cultured in standard medium: RPMI 1640 supplemented with 2 mmol/L of glutamine (Sigma-Aldrich), P+S, 15 mmol/L HEPES (Sigma-Aldrich), and with different percentages of FBS and hormones and growth factors. EE and OS cells were cultured in 0.5% FBS and a mixture of six supplements (6H) for at least 15 days. SE were cultured in 10% FBS medium. Because after 15 days, the growth rate of EE cells was very low or null, we enzymatically [trypsin-EDTA solution (Cambrex) for 2 min at 37°C] detached and seeded the cells on top of the sister semiconfluent SE dish. Cells were maintained at 37°C in a humidified atmosphere of 5% CO2. The medium was renewed twice weekly. The two cell types were easily identified by their morphology.

### Transwell cell invasion and coculture

For cell invasion assay, Transwell chamber (Corning Inc.) was coated with Matrigel and DMEM (v/v 1:8), with cultures below 80% confluence to ensure proper starvation of cells without becoming confluent during the 24- starvation period. The cell seeding concentration (5×105 cells/ml) was diluted with 150 μl serum-free medium and added to Matrigel mixture. 500 μl DMEM with 10% FBS was added to a lower compartment. Cultures were incubated for 36h. The invaded cells were stained and counted according to the manufacturers’ instructions. For cell coculture assay, an equal number of PB1 cells and RB1 cells were seeded in the upper compartment and lower compartment of Transwell chamber, respectively. Cell supernatants were collected for further assays.

### Oncosphere formation assay

For oncosphere formation assay, cells were trypsinized, counted as 5×10^5^/ml, mixed in FBS-free RPMI 1640 medium containing 20ng/ml EGF (Sigma-Aldrich), 10ng/ml FGF2 (Sigma-Aldrich), B27 (GIBCO), and ITSS (Roche), plated onto ultralow attachment plates (Corning), and incubated at 37°C with 5% CO_2_. Fresh growth medium was added every 48h. To determine the frequency of oncosphere-forming cells, one thousand tumor cells were distributed into each well in low-attachment 24-well tissue culture plates in the stem cell medium. The number of oncospheres in each well was counted on day 7. At this cell density (1,000 cells per well) in the 24-well plates, cells did not cluster together. Oncosphere formation was determined at the indicated time points by automated imaging on an inverted microscope (Axiovert 100M, Carl Zeiss).

### CCK-8 cell proliferation

Cells were counted and the concentration of the cell suspension was adjusted to 5×10^4^ cells/ml. Using serial dilution, cell suspension (100 μl) was added to each well of the 96-well microplate. 10 μl cell counting Kit-8 (CCK-8) was added to each well and reacted in a CO2 incubator for 4h. Absorbance was measured at 450 nm on a microplate reader.

### Flow cytometry

For cell apoptosis and cell cycle, 1×10^5^ cells were seeded per well in a 12-well plate for 24h and then treated with 10 μmol/L epidoxorubicin. After 24h incubation, cells were collected, stained with propidium iodide (PI) and APC-conjugated Annexin V (Apoptosis detection kit, Invitrogen), and analyzed by flow cytometry and software (BD Biosciences).

### *In vivo* phosphorylation

293 T cells were transfected with either Snail expression vector or the construct with the indicated point mutations and cotransfected with with or without catalytically active PI3K (PI3KCA). Cells were labeled with [32P]orthophosphoric acid overnight. Twenty-four hours after transfection, cell lysates were immunoprecipitated with an anti-HA monoclonal antibody (mAb) and then separated by 10% SDS-PAGE. Labeling was visualized by autoradiography with a Phosphor-Imager.

### Luciferase reporter assays

Cells in 24-well plates were transiently transfected with the indicated promoter reporter constructs and pRLTK Renilla luciferase plasmid (Promega) using Fugene 6 (Roche). Cell extracts were prepared after transfection, and luciferase activity was measured using the Dual-Luciferase Reporter Assay System (Promega).

### Protein quantitation and identification

The supernatant samples from PB1 and RB1 were in-gel digested enzymatically and analyzed by LC-MS/MS. The database search was performed using both the X!Tandem and SEQUEST algorithms. Quantification was first performed using Mascot 2.2. Using the Mascot quantification method, protein quantification was only performed on proteins identified by two or more peptides with scores above the identity threshold. Protein differential expression was also assessed at the peptide level. All peptides were used to calculate global mean and S.D. of peptide ratios (47-1/D3). The differentially expressed peptides were used to infer differentially expressed proteins.

### Statistical analysis

All statistical analyses were performed with SPSS software (SPSS, Chicago, IL, USA). Analysis of variance, Student t test and Dunnett's multiple comparison test were used to compare mean values. Data are presented as mean±SD. A p value of <.05 was defined as statistically significant.

## SUPPLEMENTARY FIGURES


