# Supplementary material for: Epithelial-mesenchymal transition induced by GRO-α-CXCR2 promotes bladder cancer recurrence after intravesical chemotherapy
Source: Oncotarget. 2017 Apr 3;8(28):45274–85. doi: 10.18632/oncotarget.16786 (PMC5542185; doi:10.18632/oncotarget.16786)
Supplement: Supplementary file 1 [file oncotarget-08-45274-s001.pdf]

Epithelial-mesenchymal transition induced by GRO-α-CXCR2 promotes bladder cancer recurrence after intravesical chemotherapy

SUPPLEMENTARY FIGURES

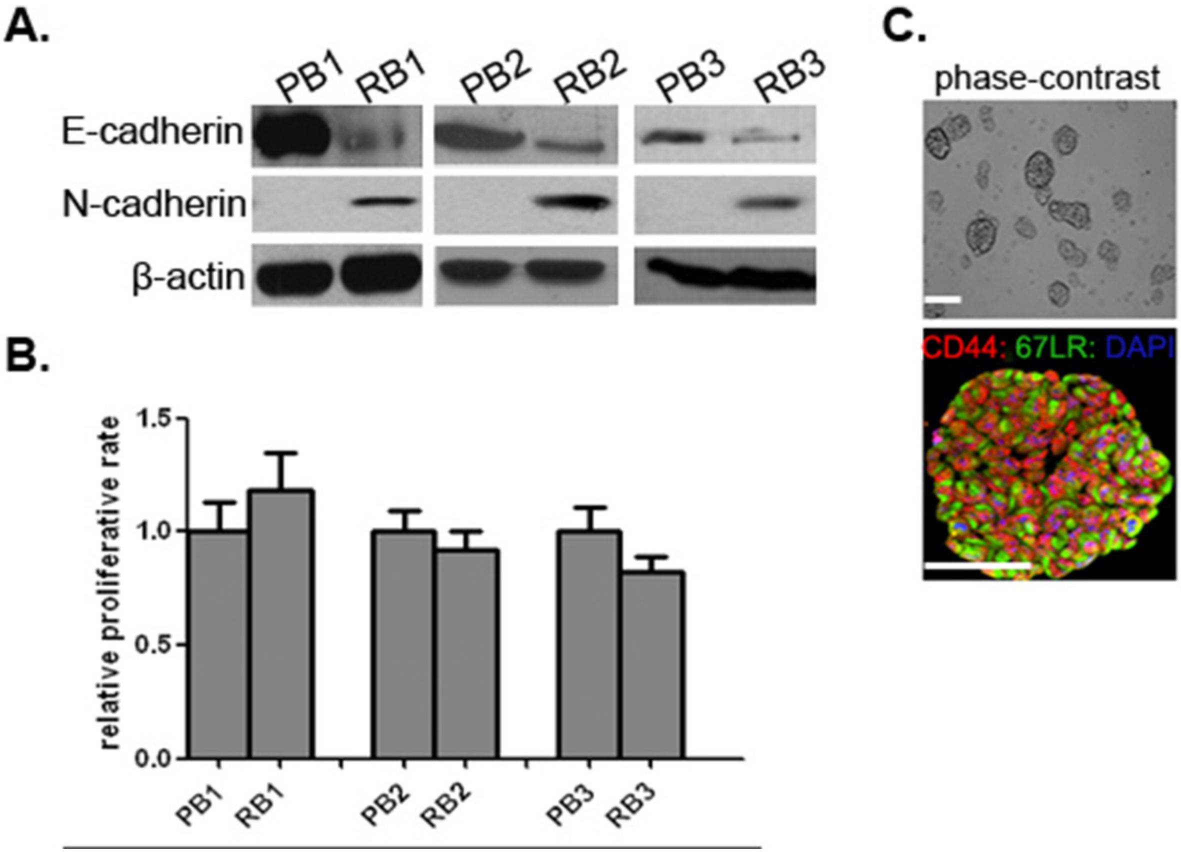

Supplementary Figure 1: Recurrent bladder cancers exhibit mesenchymal phenotypes. **(A)** The expression level of E-cadherin and N-cadherin was compared between the paired primary and recurrent bladder cancer cells. **(B)** The proliferative rate was compared between the paired primary and recurrent bladder cancer cells. **(C)** Oncospheres from RB2 cells were maintained in serum-free medium containing growth factors. Oncospheres were then co-stained with CD44 and 67LR.

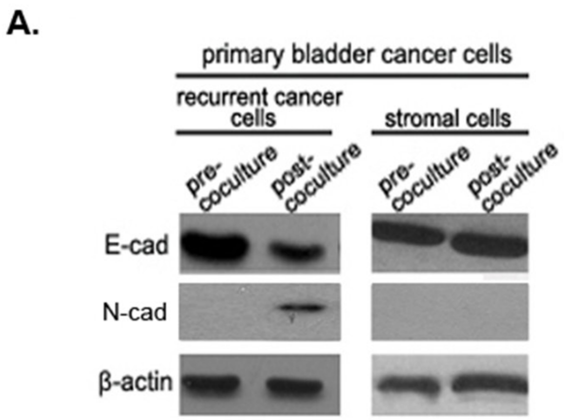

Supplementary Figure 2: EMT is initiated in recurrent bladder cancers via an autocrine manner. (A) After PB1 cells were transwell-cultured with RB1 or stromal cells as described in Figure 2A, the expression level of E-cadherin and N-cadherin was examined. **(B)** The expression level of CXCR2 was compared between the paired primary and recurrent bladder cancer cells.

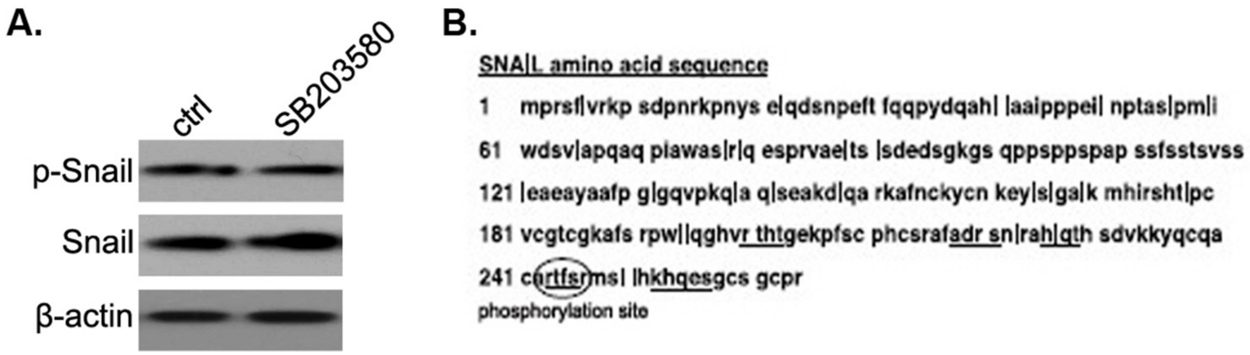

Supplementary Figure 3: GRO-α regulation of Snail phosphorylation. **(A)** RB3 cells were treated with 10μg/ml SB203580 for 48h, after which the expression level of phospho-Snail and Snail were assessed. **(B)** Snail amino acid sequence showing the possible GRO-α phosphorylation sites. Potential GRO-α phosphorylation sites are underlined and the identified GRO-α phosphorylation site is encircled.
